# Endoplasmic Reticulum–Mitochondria Contact Sites—Emerging Intracellular Signaling Hubs

**DOI:** 10.3389/fcell.2021.653828

**Published:** 2021-05-20

**Authors:** Saeko Aoyama-Ishiwatari, Yusuke Hirabayashi

**Affiliations:** ^1^Graduate School of Pharmaceutical Sciences, The University of Tokyo, Tokyo, Japan; ^2^Department of Chemistry and Biotechnology, School of Engineering, The University of Tokyo, Tokyo, Japan

**Keywords:** mitochondria, ER, organelle contact sites, mammalian protein, tether

## Abstract

It has become apparent that our textbook illustration of singular isolated organelles is obsolete. In reality, organelles form complex cooperative networks involving various types of organelles. Light microscopic and ultrastructural studies have revealed that mitochondria–endoplasmic reticulum (ER) contact sites (MERCSs) are abundant in various tissues and cell types. Indeed, MERCSs have been proposed to play critical roles in various biochemical and signaling functions such as Ca^2+^ homeostasis, lipid transfer, and regulation of organelle dynamics. While numerous proteins involved in these MERCS-dependent functions have been reported, how they coordinate and cooperate with each other has not yet been elucidated. In this review, we summarize the functions of mammalian proteins that localize at MERCSs and regulate their formation. We also discuss potential roles of the MERCS proteins in regulating multiple organelle contacts.

## Introduction

Electron microscopy (EM) studies have revealed that a significant portion of membranes from a variety of organelles are closely apposed but do not fuse. Among the membrane appositions within the range of 10–30 nm, membrane contact sites (MCSs) have been defined by the presence of proteins tethering two organelles (Scorrano et al., 2019). In recent years, it has become rapidly apparent that MCSs serve as unique intracellular platforms regulating a wide range of biochemical reactions. The mitochondria–endoplasmic reticulum (ER) contact sites (MERCSs) are the most frequently observed MCSs in many cell types, and extensive studies have revealed that MERCSs are hubs for the exchange of metabolites (Valm et al., 2017). For instance, the mitochondrial calcium uniporter (MCU) protein is activated only when the MCU complex is exposed to a high concentration of Ca^2+^ rarely reached in the cytoplasm of most cell types (1–5 μM depending on the components of the MCU complex) (Csordás et al., 2013; Patron et al., 2014; Petrungaro et al., 2015). These channel properties necessitate that the mitochondrial surface be very closely apposed to the ER surface, where Ca^2+^ is released from either inositol 1,4,5-trisphosphate (IP3) receptors (IP3Rs) and/or ryanodine receptors (RyRs) (Rizzuto et al., 1993; Cremer et al., 2020). Furthermore, lipid exchange between the ER and mitochondria is required for the coordinated synthesis of glycerophospholipids by lipid biosynthetic enzymes that localize at either the ER membrane (ERM) or the mitochondrial matrix. In the absence of vesicular transport between these organelles, transfer of intermediate lipid molecules relies on non-vesicular lipid transfer at contact sites (Vance, 1990; Petrungaro and Kornmann, 2019). In addition, MERCSs have been suggested to define sites of mitochondrial division and mitochondrial DNA (mtDNA) replication (Friedman et al., 2011; Murley et al., 2013; Lewis et al., 2016) as well as mitochondrial fusion (Guo et al., 2018; Abrisch et al., 2020). They also provide a platform for autophagosome biogenesis (Hailey et al., 2010; Hamasaki et al., 2013; Garofalo et al., 2016; Böckler and Westermann, 2014; Wu et al., 2016; Gomez-Suaga et al., 2017). In order to regulate this wide variety of functions at MERCSs, various protein complexes are specifically recruited and dynamically maintained at these unique contact sites.

In yeast, a protein complex called the ER–mitochondria encounter structure (ERMES) was identified as a molecular zipper bridging the ER and mitochondria (Kornmann et al., 2009). The ERMES complex consists of four core proteins: an ER-anchored maintenance of mitochondrial morphology 1 (Mmm1), mitochondrial distribution and morphology 10 (Mdm10) localized to the outer membrane of mitochondria (OMM), Mdm34 (Mmm2), and cytosolic Mdm12. The protein–protein interactions among these core components generate the tethering force between these two organelles. A few ERMES complex binding proteins such as a Ca^2+^-binding GTPase Gem1 (Kornmann et al., 2011; Stroud et al., 2011; Nguyen et al., 2012) and Mdm10 binding translocase of outer membrane 7 (Tom7) (Meisinger et al., 2006; Yamano et al., 2010; Becker et al., 2011; Ellenrieder et al., 2016) have been identified as the auxiliary subunits of ERMES complex. Gem1 is required for MERCS formation (Kornmann et al., 2011), whereas the function of Tom7 in regulating MERCSs is unclear.

Like Tom7, the sorting and assembly machinery (SAM) complex, which is responsible for the membrane insertion of mitochondrial outer membrane proteins, interacts with the β-barrel structure of Mdm10 at the opposite side of Mdm12 binding site (Ellenrieder et al., 2016). Given the competition between the ERMES and SAM complexes for Mdm10 binding, it is possible that MERCS formation and mitochondrial protein import are interrelated. Mmm1, Mdm12, and Mdm34 contain synaptotagmin-like mitochondrial lipid-binding protein (SMP) domains, which are homologous to the structurally well-characterized tubular lipid binding protein (TULIP) domain present in many lipid-binding proteins (I. Lee and Hong, 2006; Kopec et al., 2010). Structural analyses have shown that the SMP domain-containing proteins interact with a wide variety of glycerophospholipids (Schauder et al., 2014; AhYoung et al., 2015; Jeong et al., 2016, 2017). Thus, it has been hypothesized that ERMES might be a candidate in non-vesicular transfer of lipids between ER and mitochondria.

**FIGURE 1:**
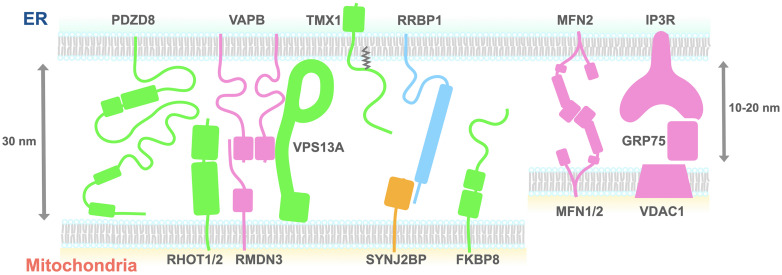
A schematic diagram of the proteins proposed to tether the ER and mitochondria. The size of each domain structure was either calculated from the structures deposited on the Protein Data Bank or estimated using the Phyre2 protein structure prediction tool. Proteins shown to be necessary (green, pink) or sufficient (yellow) for tethering the ER and mitochondria are depicted. Green indicates that tethering partners of the proteins remain to be determined. Light blue indicates that roles of the proteins need to be determined. ER, endoplasmic reticulum; FKBP8, FK506-binding protein 8; IP3R, inositol 1,4,5-trisphosphate receptor; Mfn2, mitofusin 2; PDZD8, PDZ domain-containing protein 8; RHOT1, Ras homolog family member T1; GRP75, glucose-regulated protein 75; RMDN3, regulator of microtubule dynamics protein 3; RRBP1, ribosome-binding protein 1; SYNJ2BP, synaptojanin-2-binding protein; TMX1, thioredoxin-related transmembrane protein 1; VAPB, VAMP-associated protein B; VPS13A, vacuolar protein sorting-associated protein 13 A; VDAC, voltage-dependent anion channel.

Likewise, characterizing the molecules responsible for MERCS formation in other eukaryotic cell types including multicellular organisms has been of great interest for a decade, which leads to the identification of proteins regulating this contact formation (Figure 1 and Table 1). The large variety of proteins identified indicates that the regulation of MERCSs in mammals is more complicated than in yeast. However, the coordination and dynamics of these protein complexes remain unclear and sometimes debated.

**TABLE 1:** List of MERCS-Regulating Proteins.

**Gene Name**	**Localization**	**How to assess the roles in MERCS formation**	**Interactors**	**Other Roles**	**Evolutionary conservation**	**References**
ATAD3A	Mitochondria (IMM, OMM)		-	Steroidogenesis, Cholesterol homeostasis, Cristae structure maintenance		Baudier, 2018; Issop et al., 2015
BAP31	ER		Fis1	Proapoptotic		Iwasawa et al., 2011
CISD2	Mitochondria (OMM), ER	TEM, Immunofluorescence, Ca^2+^ transfer (in WFS2-patient-derived cells) (Rouzier et al., 2017)	GIMAP5	Wolfram syndrome type 2 (WFS2)-related gene		Wang et al., 2014; Rouzier et al., 2017
CKAP4	ER	TEM, Split-GFP, Ca^2+^ transfer	VDAC2			Harada et al., 2020
Drp1	Cytoplasm, Mitochondria (OMM)	Immunofluorescence, Ca^2+^ transfer (in Mul1-deficient cells) (Prudent et al., 2015)		Mitochondrial fission		Prudent et al., 2015
EMC	ER	TEM (in yeast)	SLC25A46?	Transmembrane helix insertase	Conserved from yeast	Lahiri et al., 2014
EMD (Emerin)	Nucleus, ER		FATE1	Causal gene of Emery–Dreifuss muscular dystrophy		Doghman-Bouguerra et al., 2016
FATE1	Mitochondria (OMM), Mitochondria-associated membrane (MAM)	TEM, Immunofluorescence, Ca^2+^ transfer	EMD/Emerin?	Antiapoptotic		Doghman-Bouguerra et al., 2016
Fis1	Mitochondria (OMM)		BAP31	Mitochondrial fission, Proapoptotic		Iwasawa et al., 2011
FKBP8	Mitochondria (OMM), ER	TEM, Ca^2+^ transfer	Unknown	Antiapoptotic, Mitophagy		Kwak et al., 2020
FUNDC1	Mitochondria (OMM)	TEM (Wu et al., 2017)	IP3R2, Calnexin (under hypoxia)	Mitophagy, Mitochondrial fission, Ca^2+^ regulation		Wu et al., 2016; Wu et al., 2017
GIMAP5	MAM, ER?		CISD2			Wang et al., 2014
Gp78 (AMFR)	ER	TEM, Immunofluorescence	Mfn1/2 (ubiquitination)	E3 ubiquitin ligase, ER-associated protein degradation		Wang et al., 2015
GRP75	Cytoplasm (MAM)	Ca^2+^ transfer	IP3R, VDAC1			Szabadkai et al., 2006
INF2	ER	Immunofluorescence	Spire1C	Actin polymerization		Manor et al., 2015
IP3R	ER	TEM (Bartok et al., 2019)	Grp75	Ca^2+^ transport		Szabadkai et al., 2006; Bartok et al., 2019
Mfn1	Mitochondria (OMM)		Mfn2	Mitochondrial fusion		De Brito and Scorrano, 2008
Mfn2	Mitochondria (OMM), ER	Ca^2+^ transfer (De Brito and Scorrano, 2008) TEM (Cosson et al., 2012; Filadi et al., 2015; Naon et al., 2016)	Mfn2, Mfn1	Mitochondrial fusion		De Brito and Scorrano, 2008; Cosson et al., 2012; Filadi et al., 2018
Miga2	Mitochondria (OMM)	TEM (in fly) (Xu et al., 2020)	VAPA, VAPB	Mitochondrial fusion		Freyre et al., 2019; Xu et al., 2020
MITOL (MARCH5)	MAM, Mitochondria	Immunofluorescence, *In vitro* ER-mitochondria binding assay, Ca^2+^ transfer (Sugiura et al., 2013) SBF-SEM (Nagashima et al., 2019)	Mfn2 (ubiquitination)	E3 ubiquitin ligase		Sugiura et al., 2013; Nagashima et al., 2019
Mul1 (MAPL)	Mitochondria	Immunofluorescence, Ca^2 +^ transfer (Prudent et al., 2015) TEM, Immunofluorescence in stimulated emission depletion microscopy (STED) (Puri et al., 2019)	Mfn2 (ubiquitination), Drp1 (SUMOlylation)	E3 ubiquitin ligase		Prudent et al., 2015; Puri et al., 2019
PDZD8	ER (partially MAM)	Serial SEM, Ca^2+^ transfer,	Unknown	Resident in ER-late endosome/lysosome contacts	Potential Ortholog (Paralog) of MMM1	Hirabayashi et al., 2017
PS2	ER	Immunofluorescence, Ca^2+^ transfer	Mfn2	Causally linked to familial Alzheimer’s disease (FAD)		Filadi et al., 2016
Reep1	ER, Mitochondria	Split-RLuc8 assay (in Reep1-overexpressing cells)	Unknown	Hereditary spastic paraplegias (HSPs)-associated gene		Lim et al., 2015
RHOT1/2 (MIRO1/2)	Mitochondria (OMM)	TEM, Immunofluorescence, Ca^2+^ transfer (Modi et al., 2019)	IP3R?	Mitochondrial motility (Microtubule binding)	GEM1 in yeast	Kornmann et al., 2011; S. Lee et al., 2016; Modi et al., 2019;
RMDN3 (PTPIP51)	Mitochondria (OMM)	Ca^2+^ transfer (De vos et al., 2012) TEM, Immunofluorescence (Stoica et al., 2014)	VAPB			De vos et al., 2012; Stoica et al., 2014; Gomez-Suaga et al., 2017; Fecher et al., 2019
RRBP1	ER	TEM (Anastasia et al., 2021)	SYNJ2BP	Kinesin binding		Hung et al., 2017; Anastasia et al., 2021
SLC25A46	Mitochondria (OMM)		EMC component?	Phospholipid exchange		Janer et al., 2016
Spire1C	Mitochondria (OMM)	Immunofluorescence	INF2	Actin nucleation		Manor et al., 2015
SYNJ2BP	Mitochondria (OMM)	TEM (in SYNJ2BP-overexpressing cells)	RRBP1	Negative regulator of angiogenesis, tumor growth and metastasis		Hung et al., 2017
Tom70	Mitochondria (OMM)	SPLICS, Immunofluorescence, Ca^2+^ transfer	IP3R3	Translocase of OMM		Filadi et al., 2018
VAPB	ER	Ca^2+^ transfer (De vos et al., 2012) TEM, Immunofluorescence (Stoica et al., 2014)	RMDN3	Resident in ER–endosome contacts, ER–golgi contacts and ER–PM contacts		De vos et al., 2012; Stoica et al., 2014; Freyre et al., 2019; Xu et al., 2020
VDAC1	Mitochondria (OMM)	TEM (Bosc et al., 2020)	GRP75	Ca^2+^ transport		Szabadkai et al., 2006; Bosc et al., 2020
VDAC2	Mitochondria (OMM)		CKAP4	Ca^2+^ transport		Harada et al., 2020
VPS13A	MAM	TEM (Muñoz-Braceras et al., 2019) Immunofluorescence (Kumar et al., 2018) SPLICS (Yeshaw et al., 2019)	VAPA, VAPB	Autophagy regulation, Lipid droplet motility		Kumar et al., 2018; Muñoz-Braceras et al., 2019; Yeshaw et al., 2019.
TMX1	ER, MAM (palmitoylated)	TEM, Ca^2+^ transfer	Unknown			Raturi et al., 2016
TG2	Cytoplasm (MAM)	TEM, Proximity ligation assay, Immunofluorescence	GRP75	Posttranslational modification		D‘Eletto et al., 2018

*ATAD3A, ATPase family AAA domain-containing protein 3A; BAP31, B-cell receptor-associated protein 31; CISD2, CDGSH iron-sulfur domain-containing protein 2; CKAP4, cytoskeleton-associated protein 4; Drp1, dynamin-related protein 1; EMC, endoplasmic reticulum membrane protein complex; ER, endoplasmic reticulum; FATE1, fetal and adult testis-expressed transcript protein; Fis1, fission protein 1; FKBP8, FK506-binding protein 8; FUNDC1, FUN14 domain-containing protein 1; GIMAP5, GTPase immunity-associated protein family member 5; GRP75, glucose-regulated protein 75; IMM, inner mitochondrial membrane; INF2, Inverted formin 2; IP3R, inositol 1,4,5-trisphosphate receptor; MAM, mitochondria-associated membrane; MERCS, mitochondria–endoplasmic reticulum contact site; Mfn2, mitofusin 2; Miga2, mitoguardin 2; MITOL, Mitochondrial ubiquitin ligase; Mul1, mitochondrial ubiquitin protein ligase 1; Mul1, mitochondrial E3 ubiquitin protein ligase 1; OMM, outer membrane of mitochondria; PDZD8, PDZ domain-containing protein 8; PM, plasma membrane; PS2, presenilin 2; Reep1, receptor expression-enhancing protein 1; RHOT1, Ras homolog family member T1; RMDN3, regulator of microtubule dynamics protein 3; RRBP1, ribosome-binding protein 1; SLC25A46, solute carrier family 25 member 46; SPLICS, split GFP-based contact site sensors; SYNJ2BP, synaptojanin-2-binding protein; TEM, transmission electron microscopy; TG2, transglutaminase type 2; TMX1, thioredoxin-related transmembrane protein 1; TMX1, transmembrane protein 1; Tom7, translocase of outer membrane 7; VAPB, VAMP-associated protein B; VDAC, voltage-dependent anion channel; VPS13A, vacuolar protein sorting-associated protein 13 A.*

## Mitochondria-Endoplasmic Reticulum Contact Site-Resident Proteins Involved in the Contact Formation

### Yeast Endoplasmic Reticulum–Mitochondria Encounter Structure Complex and Functional Mammalian Homologs

Several orthologs of the ERMES complex proteins have been identified in metazoans, although until recently, only those of regulatory subunits of the complex were known. In mammals, mitochondria-localized GTPase family members Ras homolog family member T1 (RHOT1, also known as Miro1) and RHOT2 (Miro2) are identified as orthologs of Gem1. Confocal microscopy analysis in mouse embryonic fibroblasts (MEFs) showed that RHOT1 and RHOT2 double-knockout (DKO) resulted in the reduction of Mander’s coefficient between ER- and mitochondria-localized fluorescent proteins, as well as a slight decrease in the number of MERCSs identified by transmission EM (TEM) (Modi et al., 2019). Recruitment of RHOT1 to MERCS is promoted by its phosphorylation. Blocking the phosphorylation *via* a polo-like kinase 1 (PLK1) inhibitor reduced the interaction between voltage-dependent anion channel (VDAC) and IP3R, which tether the ER and mitochondria (see below) (S. Lee et al., 2016). It seems that the Tom7–SAM complex is conserved in mammals as a mitochondrial protein transport machinery; however, it remains unknown whether it is involved in MERCS formation.

Compared to the auxiliary subunits, orthologs of the core ERMES complex proteins had not been identified in metazoans because of low levels of conservation in the primary amino acid sequences. However, we recently identified the SMP domain-containing ER-resident protein called PDZ domain-containing protein 8 (PDZD8) as a functional homolog of yeast Mmm1 (Hirabayashi et al., 2017).

### Mammalian Regulators of Mitochondria–Endoplasmic Reticulum Contact Sites

The recent development of the automatic Serial Scanning EM (SSEM) enabled us to visualize the three-dimensional (3D) structures of MERCSs. This technique unequivocally demonstrated that the aforementioned MMM1 homolog PDZD8 is required for MERCS formation in human HeLa cells without any effects on the 3D architecture of ER and mitochondria. Furthermore, dual-color Ca^2+^ imaging showed that ER–mitochondria tethering mediated by PDZD8 is essential for Ca^2+^ flux from the ER to the mitochondria in NIH3T3 cells and in mouse cortical pyramidal neurons (Hirabayashi et al., 2017). A recent *in vitro* assay suggested that SMP and PDZ domains of PDZD8 extract glycerophospholipids and ceramides, as well as cholesterol, albeit with low efficiency (Shirane et al., 2020). Thus, PDZD8 is potentially capable of transferring lipids in MCSs. Further studies are required to reveal the mitochondrial binding partner of PDZD8.

Mitochondria fusion protein mitofusin 2 (Mfn2) is the most intensively studied yet controversial ER–mitochondria tethering protein. It has been proposed that ER-localized Mfn2 engages in homotypic and heterotypic complexes with OMM localizing Mfn2 or Mnf1, respectively (De Brito and Scorrano, 2008). However, the role of Mfn2 complex in MERCS formation has been under intense debate (Filadi et al., 2018). Indeed, a recent study using split green fluorescent protein (GFP)-based contact site sensors (SPLICSs) showed that Mfn2 depletion increased and decreased the number of ER–mitochondria contact sites detected by SPLICS having spacers corresponding to short (8–10 nm) and long (40–50 nm) membrane distances, respectively (Cieri et al., 2018). Considering that the loss of Mfn1/2 complex increased the average distance between the two membranes at MERCSs only to 18 nm (De Brito and Scorrano, 2008), it is possible that the Mfn complex fine-tunes the membrane distance at MERCSs. It remains to be elucidated in what context and how Mfn2 controls MERCSs, as well as if it is related to its role in OMM fusion.

Consistent with the observation that mitochondrial fission occurs at MERCSs, the mitochondrial fission protein 1 (Fis1) is proposed to reside at MERCSs *via* the interaction with an ER-resident protein B-cell receptor-associated protein 31 (BAP31) (Iwasawa et al., 2011), although it remains uninvestigated whether the complex impacts MERCS formation. Also, the mitochondrial fission GTPase dynamin-related protein 1 (Drp1) has been shown to functionally stabilize MERCSs through mitochondrial E3 ubiquitin protein ligase 1 (Mul1, also known as MAPL)-dependent SUMOylation (Prudent et al., 2015).

The OMM-localized protein Spire1C, one of the splicing isoforms of Spire1, is yet another protein regulating mitochondrial fission at MERCSs. Spire1C interacts with the ER-localized isoform of Inverted formin 2 (INF2) *via* its kinase non-catalytic C-lobe domain (KIND) (Manor et al., 2015). The overexpression of KIND-deleted Spire1C decreased the overlap between the ER and mitochondria as identified by immunofluorescence imaging in light microscopy.

The yeast two-hybrid screening identified VDAC1–glucose-regulated protein (GRP)75–IP3R complex as a linker between the ER and mitochondria (Szabadkai et al., 2006). The OMM channel VDAC1 mediates Ca^2+^ channeling from microdomains with high Ca^2+^ concentration generated by the opening of the ER-resident Ca^2+^-release channel IP3Rs to the intermembrane space (Gincel et al., 2001; Rapizzi et al., 2002). IP3R2 is reported to interact not only with VDAC but also with the OMM-localized protein FUN14 domain-containing protein 1 (FUNDC1) at least in cardiac myocytes (S. Wu et al., 2017). Grp75 is a cytosolic regulator of the IP3R–VDAC complex that promotes the interaction between the channels to increase the efficiency of mitochondrial Ca^2+^ uptake (Szabadkai et al., 2006). The interaction between Grp75 and a MERCS-localized protein transglutaminase type 2 (TG2) has also been suggested to contribute to MERCS formation (D‘Eletto et al., 2018). Inferred from the 3D structure of each protein, the size of VDAC1–GRP75–IP3R complex is around 15 nm. Given that the triple-KO (TKO) of IP3R isoforms decreased more specifically the tight contact sites where the two membranes are less than 20 nm apart (Bartok et al., 2019), it is likely that this complex brings the ER and mitochondria in particularly close proximity. Tom70 was also reported to interact with IP3R3 to recruit it to the proximity of mitochondria, which results in promoting Ca^2+^ transfer from the ER to mitochondria (Filadi et al., 2018).

Another protein complex identified by the yeast two-hybrid screening is the VAMP-associated protein B (VAPB)–regulator of microtubule dynamics protein 3 (RMDN3, also called PTPIP51) complex (De vos et al., 2012). The quantification of the single-plane TEM images revealed that depletion of either VAPB or RMDN3 reduced MERCSs in human HEK293 cells (Stoica et al., 2014). The role of RMDN3 in MERCS formation was also shown in cerebellar Purkinje cells (Fecher et al., 2019). Knocking down either VAPB or RMDN3 caused a significant delay but only slight decrease in the mitochondrial Ca^2+^ uptake (De vos et al., 2012) and stimulated the induction of autophagy flux (Gomez-Suaga et al., 2017). Recently, the OMM protein mitoguardin 2 (Miga2) was also reported to interact with VAPA and VAPB (Freyre et al., 2019) and proposed to increase MERCS formation at least in flies (Xu et al., 2020). VAP-interacting protein vacuolar protein sorting-associated protein 13 A (VPS13A) has also been reported to localize to MERCSs and participate in their stabilization (Kumar et al., 2018; Muñoz-Braceras et al., 2019; Yeshaw et al., 2019). Interestingly, a recent study showed that RMDN3 recruits the oxysterol-binding protein (OSBP)-related proteins ORP5 and ORP8, which transfer phosphatidylinositol (PI) and phosphatidylserine (PS) at ER–plasma membrane (PM) contact sites (Chung et al., 2015; Moser von Filseck et al., 2015), to MERCSs (Galmes et al., 2016). Consistent with that, ORP5 and ORP8 are proposed to mediate PS transport, likely *via* the non-vesicular lipid transfer, at MERCSs (Rochin et al., 2019).

The OMM-localized fetal and adult testis-expressed transcript protein (FATE1) known as an antiapoptotic protein may also contribute to ER–mitochondria tethering. Overexpression of FATE1 partly decreased MERCSs as identified by TEM and confocal microscopy and also reduced Ca^2+^ uptake by mitochondria (Doghman-Bouguerra et al., 2016). EMD/Emerin is a potential interactor of FATE1 in the ER; however, the role of EMD in MERCS formation has not been investigated.

Posttranslational palmitoylation is found in several proteins localizing at MERCSs. The heterozygous KO of the redox-sensitive oxidoreductase thioredoxin-related transmembrane protein 1 (TMX1) decreased the average length of MERCSs as analyzed by TEM (Raturi et al., 2016). Importantly, the recruitment of TMX1 to MERCSs requires palmitoylation at the cytosolic domain (Roth et al., 2009; Lynes et al., 2012; Figure 1). Recruitment of the ER chaperone calnexin to MERCSs also requires palmitoylation (Lynes et al., 2012). While the palmitoylation of TMX1 is required for proper Ca^2+^ uptake from the ER to mitochondria, the ER-resident protein cytoskeleton-associated protein 4 (CKAP4) requires palmitoylation for sequestering VDAC2 from IP3R, which results in a decrease of MERCSs (Harada et al., 2020).

A proteomic analysis of the intersection between OMM and ERM-resident proteins obtained from ascorbate peroxidase (APEX)-mediated proximity biotinylation and subsequent mass spectrometry (MS) analysis identified the synaptojanin-2-binding protein (SYNJ2BP)–ribosome-binding protein 1 (RRBP1) complex as a potential tether that specifically regulates mitochondria–rough ER contact sites (Hung et al., 2017). TEM analysis showed that SYNJ2BP overexpression increased contacts between mitochondria and rough ER but not between mitochondria and smooth ER. In line with this, a recent report showed that RRBP1 is resident in mitochondria–rough ER contacts in mouse liver and works as a regulator of these contacts (Anastasia et al., 2021). The list of proteins identified in the intersection confirmed the localization of known MERCS proteins, even though many MERCS proteins, such as PDZD8, VAPs, and IP3Rs, listed in both ERM and OMM proteins are excluded from the intersection list because they also localized outside of MERCSs.

Recently, two split-pair proximity labeling enzymes, Contact-ID and Split-TurboID, were applied for the direct mapping of proteins localizing at MERCSs (Cho et al., 2020; Kwak et al., 2020). Although these methods are less sensitive than other methods, proteins identified with these methods are reliably localized to MERCS. Contact-ID identified FK506-binding protein 8 (FKBP8) as a novel MERCS-localizing protein. Indeed, knocking down FKBP8 reduces MERCSs in the TEM analysis and also diminishes mitochondrial Ca^2+^ uptake (Kwak et al., 2020).

### Disease Association of Mitochondria–Endoplasmic Reticulum Contact Site Proteins

Genome-wide association studies have identified numerous gene mutations associated with neurological diseases. Among those, a significant number of mutations are found in genes related to MERCS formation, such as Mfn2, receptor expression-enhancing protein 1 (Reep1), solute carrier family 25 member 46 (SLC25A46), ATPase family AAA domain-containing protein 3A (ATAD3A), and CDGSH iron-sulfur domain-containing protein 2 (CISD2). Mutations in a genomic region coding an ER-resident protein Reep1 is associated with hereditary spastic paraplegias (HSPs) and distal hereditary motor neuropathy. A split-Renilla Luciferase 8 (RLuc8) reassembly assay suggested that Reep1 facilitates MERCS formation (Lim et al., 2015). The disease-associated *Reep1* mutations impair REEP1’s ability to facilitate MERCSs, implying the relationship between HSP pathology and Reep1 function in MERCS formation (Lim et al., 2015).

A recent study using fibroblasts obtained from a patient suffering from Leigh syndrome identified a homozygous missense mutation in the genomic region coding for the mitochondrial protein SLC25A46. The study also proposed that SLC25A46 interacts with the conserved ER membrane protein complex (EMC) at MERCSs (Janer et al., 2016). Interestingly, EMC is suggested to be involved in MERCS formation in yeast (Lahiri et al., 2014). Moreover, it was shown that loss of SLC25A46 altered mitochondrial phospholipid composition, implying that SLC25A46 also plays a role in promoting lipid transfer at MERCSs (Janer et al., 2016). Further studies are still required to elucidate the function of SLC25A46 and EMC in MERCS formation in mammalian cells.

Another disease-associated gene, the inner mitochondrial membrane (IMM)-localized protein ATAD3A, has also been proposed to participate in MERCS formation (Issop et al., 2015; Baudier, 2018). Although the exact structure of ATAD3A remains unknown, a recent report proposed that the N-terminus of ATAD3A may insert into the OMM and associate with the ER (Issop et al., 2015). Thereby, ATAD3A may regulate the interactions among the IMM, OMM, and ERM.

Finally, another disease-related gene coding for a MERCS-regulating protein is CISD2, which is a causative gene associated with Wolfram syndrome. A recent study showed that MERCS formation and Ca^2+^ uptake in mitochondria are upregulated in patient-derived fibroblasts (Rouzier et al., 2017). In mouse white adipose tissues and fibroblasts, CISD2 is proposed to interact with GTPase immunity-associated protein (IMAP) family member 5 (GIMAP5) at MERCSs and modulates mitochondrial Ca^2+^ uptake (C. H. Wang et al., 2014).

## Mitochondria–Endoplasmic Reticulum Contact Site Proteins at Other Organelle Contact Sites

The recent development of high-speed super-resolution microscopy revealed that the ER contacts various organelles (Valm et al., 2017). Interestingly, some of the MERCSs regulating ER-resident proteins are also found at other organelle contact sites, which implies that those proteins mediate crosstalk among multiple types of organelle contacts.

A major common function of the membrane contact sites is non-vesicular lipid transfer. In this regard, VAPA and VAPB play essential roles in diverse contact sites by transferring phospholipids and ceramides. VAP proteins interact with FFAT motifs of protein partners located on the opposing membrane or the ERM (H. Wu et al., 2018). Besides RMDN3 (PTPIP51) at MERCSs, VAP proteins form complexes with proteins such as Nir2, ceramide transferase 1 (CERT), and OSBP at the ER–Golgi contacts, and OSBP, StAR-related lipid transfer protein 3 (STARD3), Protrudin, and ORP1L at the ER–endolysosome contacts.

As mentioned above, ORP5 and ORP8, which promote the exchange of PI and PS at EM–PM contacts, also localize to MERCSs (Galmes et al., 2016). Further ORP5 is suggested to localize to ER–lipid droplet contacts and regulates the exchange of phosphatidylinositol-4-phosphate (PI4P) and PS at these contacts (Du et al., 2020).

Another example of a lipid-binding MERCS protein found in other organelle contact sites is PDZD8. Although it is still controversial if PDZD8 localizes to lysosomal-associated membrane protein 1 (LAMP1)-positive lysosomes, overexpressed PDZD8 directly interacts with Protrudin and GTP-bound Rab7, both of which localize to late endosomes (Guillén-Samander et al., 2019; Elbaz-Alon et al., 2020; Shirane et al., 2020). Interestingly, overexpressed PDZD8 and Rab7 colocalize at the three-way junction of ER, endosomes, and mitochondria, thereby inducing the association of the endosome and mitochondria. Furthermore, a recent study indicates that PDZD8 participates in the VAP complex (Cabukusta et al., 2020), which implies PDZD8’s roles in multiple different organelle contact sites. Since overexpression of MERCS proteins can disrupt their localization and therefore their functions, future studies will need to elucidate the location of *endogenous* PDZD8 among those contact sites. This statement is true for most proteins studied at MERCSs. The rapid development of clustered regularly interspaced short palindromic repeats (CRISPR)-Cas9-mediated knockin strategies (as used for PDZD8 in Hirabayashi et al., 2017) in various cell types will be a key step in cell biology of MERCS protein complexes.

VPS13A is yet another lipid-binding MERCS protein that localizes to additional MCSs. Yeast Vps13 resides at mitochondria–vacuole contacts (vacuole and mitochondria patches; v-CLAMPs) and ER–vacuole contacts (nuclear–vacuole junction; NVJ) and has a redundant role with the ERMES complex. It has been hypothesized that Vps13 creates an alternative lipid transport route between the ER and mitochondria through other organelles (Lang et al., 2015; Petrungaro and Kornmann, 2019). Consistent with this idea, the mammalian orthologs of Vps13, VPS13A, and VPS13C, have been reported to possess the ability to transfer glycerophospholipids between membranes *in vitro* (Kumar et al., 2018). Furthermore, VPS13A has been reported to localize at mitochondria–endosome/lysosome contacts and ER–lipid droplet contacts as well as MERCSs, whereas VPS13C is distributed to ER–endosome contacts and ER–lipid droplet contacts, loss of which causes mitochondrial dysfunction (Lesage et al., 2016; Kumar et al., 2018). These reports imply the function of VPS13 family proteins in lipid transport at multiple MCSs, although they also play distinct roles at each contact site, such as MERCS formation, autophagy induction, and regulation of lipid droplet motility (Muñoz-Braceras et al., 2015, 2019; Kumar et al., 2018; Yeshaw et al., 2019). Since both VPS13A and VPS13C are recruited to the ER *via* the FFAT motif (Kumar et al., 2018; Yeshaw et al., 2019), it is plausible that their localization at ER–other organelle contacts might be regulated through the interaction with VAPs. It also has been shown that VPS13A interacts with Rab7 (Muñoz-Braceras et al., 2019), which may result in VPS13A’s recruitment to mitochondria–endosome contacts.

Mitochondria also form contact sites with organelles other than the ER. Several MERCS-localized mitochondrial proteins have also been reported to reside at other organelle contact sites. Mfn2 localizes at the contact sites between mitochondria and the lysosome-related organelle of pigment cells melanosome (Daniele et al., 2014). Considering that Fzo1, a yeast homolog of Mfn, is suggested to reside at mitochondria–peroxisome contacts (Shai et al., 2018), Mfns might participate in the contact formation between mitochondria and other various organelles. The mitochondria–lysosome contacts mark at the site of mitochondrial fission. At this fission site, Fis1 recruits the Rab7 GTPase-activating protein TBC1 domain family member 15 (TBC1D15), which results in untethering of the contacts (Wong et al., 2018). This suggests that Fis1 localizes at the mitochondria–lysosome contact sites, as well as at MERCSs.

## Conclusion

In recent years, owing to advances in microscopy and the development of new biochemical tools, the list of proteins involved in the regulation of MERCSs has been dramatically expanded. Given that PDZD8 remains the only identified mammalian ortholog of the ERMES core subunits (Mmm1), it is conceivable that the mammalian ER and mitochondria tethering protein complexes have not directly evolved from the yeast ERMES complex. Therefore, MERCSs might have evolved various cell type-specific roles in mammals, which are just beginning to be explored. Provided that the properties of MERCS proteins, such as domain structure, size, and localization, are quite diverse, it is plausible to assume that they work at different subdomains of MERCSs, different steps of MERCS formation, or in different cell types. This complex regulation of MERCSs might be required for the precise control of biochemical reactions in response to the various cellular demands unique to each cell type. Since many MERCS proteins also reside at other organelle contact sites, investigation of the dynamic localization of endogenous, as opposed to overexpressed, proteins in a variety of cellular contexts will improve our understanding of the complex spatiotemporal regulation of MERCSs and pave the way to reveal the physiological roles of these contact sites.

## Author Contributions

Both authors wrote the manuscript. Both authors contributed to the article and approved the submitted version.

## Conflict of Interest

The authors declare that the research was conducted in the absence of any commercial or financial relationships that could be construed as a potential conflict of interest.

## References

[B1] Abrisch R. G. Gumbin S. C. Wisniewski B. T. Lackner L. L. Voeltz G. K. (2020). Fission and fusion machineries converge at ER contact sites to regulate mitochondrial morphology. *J. Cell Biol.* 219:e201911122. 10.1083/jcb.201911122 32328629 PMC7147108

[B2] AhYoung A. P. Jiang J. Zhang J. Dang X. K. Loo J. A. Zhou Z. H. (2015). Conserved SMP domains of the ERMES complex bind phospholipids and mediate tether assembly. *Proc. Natl. Acad. Sci. U S A.* 112 E3179–E3188. 10.1073/pnas.1422363112 26056272 PMC4485115

[B3] Anastasia I. Ilacqua N. Raimondi A. Lemieux P. Ghandehari-Alavijeh R. Faure G. (2021). Mitochondria-rough-er contacts in the liver regulate systemic lipid homeostasis. *Cell Rep.* 34:108873. 10.1016/j.celrep.2021.108873 33730569

[B4] Bartok A. Weaver D. Golenár T. Nichtova Z. Katona M. Bánsághi S. (2019). IP3 receptor isoforms differently regulate ER-mitochondrial contacts and local calcium transfer. *Nat. Commun.* 10:3726. 10.1038/s41467-019-11646-3 31427578 PMC6700175

[B5] Baudier J. (2018). ATAD3 proteins: brokers of a mitochondria-endoplasmic reticulum connection in mammalian cells. *Biol. Rev.* 93 827–844. 10.1111/brv.12373 28941010

[B6] Becker T. Wenz L. S. Thornton N. Stroud D. Meisinger C. Wiedemann N. (2011). Biogenesis of mitochondria: dual role of Tom7 in modulating assembly of the preprotein translocase of the outer membrane. *J. Mol. Biol.* 405 113–124. 10.1016/j.jmb.2010.11.002 21059357

[B7] Böckler S. Westermann B. (2014). Mitochondrial ER contacts are crucial for mitophagy in yeast. *Dev. Cell.* 28 450–458. 10.1016/j.devcel.2014.01.012 24530295

[B8] Bosc C. Broin N. Fanjul M. Saland E. Farge T. Courdy C. (2020). Autophagy regulates fatty acid availability for oxidative phosphorylation through mitochondria-endoplasmic reticulum contact sites. *Nat. Commun.* 11:4056. 10.1038/s41467-020-17882-2 32792483 PMC7426880

[B9] Cabukusta B. Berlin I. van Elsland D. M. Forkink I. Spits M. de Jong A. W. M. (2020). Human VAPome analysis reveals MOSPD1 and MOSPD3 as membrane contact site proteins interacting with FFAT-Related FFNT motifs. *Cell Rep.* 33:108475. 10.1016/j.celrep.2020.108475 33296653

[B10] Cho K. F. Branon T. C. Rajeev S. Svinkina T. Udeshi N. D. Thoudam T. (2020). Split-TurboID enables contact-dependent proximity labeling in cells. *Proc. Natl. Acad. Sci. U S A.* 117 12143–12154. 10.1073/pnas.1919528117 32424107 PMC7275672

[B11] Chung J. Torta F. Masai K. Lucast L. Czapla H. Tanner L. B. (2015). PI4P/phosphatidylserine countertransport at ORP5- and ORP8-mediated ER - plasma membrane contacts. *Science* 349 428–432. 10.1126/science.aab1370 26206935 PMC4638224

[B12] Cieri D. Vicario M. Giacomello M. Vallese F. Filadi R. Wagner T. (2018). SPLICS: a split green fluorescent protein-based contact site sensor for narrow and wide heterotypic organelle juxtaposition. *Cell Death Differ.* 25 1131–1145. 10.1038/s41418-017-0033-z 29229997 PMC5988678

[B13] Cosson P. Marchetti A. Ravazzola M. Orci L. (2012). Mitofusin-2 independent juxtaposition of endoplasmic reticulum and mitochondria: an ultrastructural study. *PLoS One.* 7:e46293. 10.1371/journal.pone.0046293 23029466 PMC3460865

[B14] Cremer T. Neefjes J. Berlin I. (2020). The journey of Ca^2+^ through the cell – pulsing through the network of ER membrane contact sites. *J. Cell Sci.* 133:jcs249136. 10.1242/jcs.249136 33376155

[B15] Csordás G. Golenár T. Seifert E. L. Kamer K. J. Sancak Y. Perocchi F. (2013). MICU1 controls both the threshold and cooperative activation of the mitochondrial Ca^2+^ uniporter. *Cell. Metabol.* 17 976–987. 10.1016/j.cmet.2013.04.020 23747253 PMC3722067

[B16] Daniele T. Hurbain I. Vago R. Casari G. Raposo G. Tacchetti C. (2014). Mitochondria and melanosomes establish physical contacts modulated by Mfn2 and involved in organelle biogenesis. *Curr. Biol.* 24 393–403. 10.1016/j.cub.2014.01.007 24485836

[B17] De Brito O. M. Scorrano L. (2008). Mitofusin 2 tethers endoplasmic reticulum to mitochondria. *Nature* 456 605–610. 10.1038/nature07534 19052620

[B18] De vos K. J. Mórotz G. M. Stoica R. Tudor E. L. Lau K. F. Ackerley S. (2012). VAPB interacts with the mitochondrial protein PTPIP51 to regulate calcium homeostasis. *Hum. Mol. Genet.* 21 1299–1311. 10.1093/hmg/ddr559 22131369 PMC3284118

[B19] Doghman-Bouguerra M. Granatiero V. Sbiera S. Sbiera I. Lacas-Gervais S. Brau F. (2016). FATE 1 antagonizes calcium- and drug-induced apoptosis by uncoupling ER and mitochondria. *EMBO Rep.* 17 1264–1280. 10.15252/embr.201541504 27402544 PMC5007562

[B20] Du X. Zhou L. Aw Y. C. Mak H. Y. Xu Y. Rae J. (2020). ORP5 localizes to ER-lipid droplet contacts and regulates the level of PI(4)P on lipid droplets. *J. Cell Biol.* 219:e201905162. 10.1083/jcb.201905162 31653673 PMC7039201

[B21] D‘Eletto M. Rossin F. Occhigrossi L. Farrace M. G. Faccenda D. Desai R. (2018). Transglutaminase type 2 regulates ER-mitochondria contact sites by interacting with GRP75. *Cell. Rep.* 25 3573–3581. 10.1016/j.celrep.2018.11.094 30590033

[B22] Elbaz-Alon Y. Guo Y. Segev N. Harel M. Quinnell D. E. Geiger T. (2020). PDZD8 interacts with protrudin and Rab7 at ER-late endosome membrane contact sites associated with mitochondria. *Nat. Commun.* 11:3645. 10.1038/s41467-020-17451-7 32686675 PMC7371716

[B23] Ellenrieder L. Opaliłski Ł Becker L. Krüger V. Mirus O. Straub S. P. (2016). Separating mitochondrial protein assembly and endoplasmic reticulum tethering by selective coupling of Mdm10. *Nat. Commun.* 7:13021. 10.1038/ncomms13021 27721450 PMC5476798

[B24] Fecher C. Trovò L. Müller S. A. Snaidero N. Wettmarshausen J. Heink S. (2019). Cell-type-specific profiling of brain mitochondria reveals functional and molecular diversity. *Nat. Neurosci.* 22 1731–1742. 10.1038/s41593-019-0479-z 31501572

[B25] Filadi R. Greotti E. Pizzo P. (2018). Highlighting the endoplasmic reticulum-mitochondria connection: focus on mitofusin 2. *Pharmacol. Res.* 128 42–51. 10.1016/j.phrs.2018.01.003 29309902

[B26] Filadi R. Greotti E. Turacchio G. Luini A. Pozzan T. Pizzo P. (2015). Mitofusin 2 ablation increases endoplasmic reticulum-mitochondria coupling. *Proc. Natl. Acad. Sci. U S A.* 112 E2174–E2181. 10.1073/pnas.1504880112 25870285 PMC4418914

[B27] Filadi R. Greotti E. Turacchio G. Luini A. Pozzan T. Pizzo P. (2016). Presenilin 2 modulates endoplasmic reticulum-mitochondria coupling by tuning the antagonistic effect of mitofusin 2. *Cell Rep.* 15 2226–2238. 10.1016/j.celrep.2016.05.013 27239030

[B28] Filadi R. Leal N. S. Schreiner B. Rossi A. Dentoni G. Pinho C. M. (2018). TOM70 sustains cell bioenergetics by promoting IP3R3-mediated ER to mitochondria Ca^2+^ transfer. *Curr. Biol.* 28 369–382. 10.1016/j.cub.2017.12.047 29395920

[B29] Freyre C. A. C. Rauher P. C. Ejsing C. S. Klemm R. W. (2019). MIGA2 links mitochondria, the ER, and lipid droplets and promotes De novo lipogenesis in adipocytes. *Mol. Cell.* 76 811–825. 10.1016/j.molcel.2019.09.011 31628041

[B30] Friedman J. R. Lackner L. L. West M. DiBenedetto J. R. Nunnari J. Voeltz G. K. (2011). ER tubules mark sites of mitochondrial division. *Science* 334 358–362. 10.1126/science.1207385 21885730 PMC3366560

[B31] Galmes R. Houcine A. Vliet A. R. Agostinis P. Jackson C. L. Giordano F. (2016). ORP5/ORP8 localize to endoplasmic reticulum–mitochondria contacts and are involved in mitochondrial function. *EMBO Rep.* 17 800–810. 10.15252/embr.201541108 27113756 PMC5278607

[B32] Garofalo T. Matarrese P. Manganelli V. Marconi M. Tinari A. Gambardella L. (2016). Evidence for the involvement of lipid rafts localized at the ER-mitochondria associated membranes in autophagosome formation. *Autophagy* 12 917–935. 10.1080/15548627.2016.1160971 27123544 PMC4922444

[B33] Gincel D. Zaid H. Shoshan-Barmatz V. (2001). Calcium binding and translocation by the voltage-dependent anion channel: a possible regulatory mechanism in mitochondrial function. *Biochem. J.* 358 147–155. 11485562 10.1042/0264-6021:3580147 PMC1222042

[B34] Gomez-Suaga P. Paillusson S. Stoica R. Noble W. Hanger D. P. Miller C. C. J. (2017). The ER-mitochondria tethering complex VAPB-PTPIP51 regulates autophagy. *Curr. Biol.* 27 371–385. 10.1016/j.cub.2016.12.038 28132811 PMC5300905

[B35] Guillén-Samander A. Bian X. de Camilli P. (2019). PDZD8 mediates a Rab7-dependent interaction of the ER with late endosomes and lysosomes. *Proc. Natl. Acad. Sci. U S A.* 116 22619–22623. 10.1073/pnas.1913509116 31636202 PMC6842579

[B36] Guo Y. Li D. Zhang S. Yang Y. Liu J. J. Wang X. (2018). Visualizing intracellular organelle and cytoskeletal interactions at nanoscale resolution on millisecond timescales. *Cell* 175 1430–1442. 10.1016/j.cell.2018.09.057 30454650

[B37] Hailey D. W. Rambold A. S. Satpute-Krishnan P. Mitra K. Sougrat R. Kim P. K. (2010). Mitochondria supply membranes for autophagosome biogenesis during starvation. *Cell* 141 656–667. 10.1016/j.cell.2010.04.009 20478256 PMC3059894

[B38] Hamasaki M. Furuta N. Matsuda A. Nezu A. Yamamoto A. Fujita N. (2013). Autophagosomes form at ER-mitochondria contact sites. *Nature* 495 389–393. 10.1038/nature11910 23455425

[B39] Harada T. Sada R. Osugi Y. Matsumoto S. Matsuda T. Hayashi-Nishino M. (2020). Palmitoylated CKAP4 regulates mitochondrial functions through an interaction with VDAC2 at ER-mitochondria contact sites. *J. Cell. Sci.* 133:jcs249045. 10.1242/jcs.249045 33067255

[B40] Hirabayashi Y. Kwon S. K. Paek H. Pernice W. M. Paul M. A. Lee J. (2017). ER-mitochondria tethering by PDZD8 regulates Ca^2+^ dynamics in mammalian neurons. *Science* 358 623–630. 10.1126/science.aan6009 29097544 PMC5818999

[B41] Hung V. Lam S. S. Udeshi N. D. Svinkina T. Guzman G. Mootha V. K. (2017). Proteomic mapping of cytosol-facing outer mitochondrial and ER membranes in living human cells by proximity biotinylation. *Elife* 6:e24463. 10.7554/eLife.24463 28441135 PMC5404927

[B42] Issop L. Fan J. Lee S. Rone M. B. Basu K. Mui J. (2015). Mitochondria-associated membrane formation in hormone-stimulated leydig cell steroidogenesis: role of ATAD3. *Endocrinology* 156 334–345. 10.1210/en.2014-1503 25375035

[B43] Iwasawa R. Mahul-Mellier A. L. Datler C. Pazarentzos E. Grimm S. (2011). Fis1 and Bap31 bridge the mitochondria-ER interface to establish a platform for apoptosis induction. *EMBO J.* 30 556–568. 10.1038/emboj.2010.346 21183955 PMC3034017

[B44] Janer A. Prudent J. Paupe V. Fahiminiya S. Majewski J. Sgarioto N. (2016). SLC25A46 is required for mitochondrial lipid homeostasis and cristae maintenance and is responsible for Leigh syndrome. *EMBO Mol. Med.* 8 1019–1038. 10.15252/emmm.201506159 27390132 PMC5009808

[B45] Jeong H. Park J. Jun Y. Lee C. (2017). Crystal structures of Mmm1 and Mdm12–Mmm1 reveal mechanistic insight into phospholipid trafficking at ER-mitochondria contact sites. *Proc. Natl. Acad. Sci. U S A.* 114 E9502–E9511. 10.1073/pnas.1715592114 29078410 PMC5692604

[B46] Jeong H. Park J. Lee C. (2016). Crystal structure of Mdm12 reveals the architecture and dynamic organization of the ERMES complex. *EMBO Rep.* 17 1857–1871. 10.15252/embr.201642706 27821511 PMC5283602

[B47] Kopec K. O. Alva V. Lupas A. N. (2010). Homology of SMP domains to the TULIP superfamily of lipid-binding proteins provides a structural basis for lipid exchange between ER and mitochondria. *Bioinformatics* 26 1927–1931. 10.1093/bioinformatics/btq326 20554689 PMC2916718

[B48] Kornmann B. Currie E. Collins S. R. Schuldiner M. Nunnari J. Weissman J. S. (2009). An ER-mitochondria tethering complex revealed by a synthetic biology screen. *Science* 325 477–481. 10.1126/science.1175088 19556461 PMC2933203

[B49] Kornmann B. Osman C. Walter P. (2011). The conserved GTPase Gem1 regulates endoplasmic reticulum-mitochondria connections. *Proc. Natl. Acad. Sci. U S A.* 108 14151–14156. 10.1073/pnas.1111314108 21825164 PMC3161550

[B50] Kumar N. Leonzino M. Hancock-Cerutti W. Horenkamp F. A. Li P. Q. Lees J. A. (2018). VPS13A and VPS13C are lipid transport proteins differentially localized at ER contact sites. *J. Cell. Biol.* 217 3625–3639. 10.1083/JCB.201807019 30093493 PMC6168267

[B51] Kwak C. Shin S. Park J. S. Jung M. My Nhung T. T. Kang M. G. (2020). Contact-ID, a tool for profiling organelle contact sites, reveals regulatory proteins of mitochondrial-associated membrane formation. *Proc. Natl. Acad. Sci. U S A.* 117 12109–12120. 10.1073/pnas.1916584117 32414919 PMC7275737

[B52] Lahiri S. Chao J. T. Tavassoli S. Wong A. K. O. Choudhary V. Young B. P. (2014). A conserved endoplasmic reticulum membrane protein complex (EMC) facilitates phospholipid transfer from the ER to mitochondria. *PLoS Biol.* 12:1001969. 10.1371/journal.pbio.1001969 25313861 PMC4196738

[B53] Lang A. B. John Peter A. T. A. T. Walter P. Kornmann B. (2015). ER-mitochondrial junctions can be bypassed by dominant mutations in the endosomal protein Vps13. *J. Cell. Biol.* 210 883–890. 10.1083/jcb.201502105 26370498 PMC4576869

[B54] Lee I. Hong W. (2006). Diverse membrane-associated proteins contain a novel SMP domain. *FASEB J.* 20 202–206. 10.1096/fj.05-4581hyp 16449791

[B55] Lee S. Lee K. S. Huh S. Liu S. Lee D. Y. Hong S. H. (2016). Polo kinase phosphorylates miro to control ER-mitochondria contact sites and mitochondrial Ca^2+^ homeostasis in neural stem cell development. *Dev. Cell.* 37 174–189. 10.1016/j.devcel.2016.03.023 27093086 PMC4839004

[B56] Lesage S. Drouet V. Majounie E. Deramecourt V. Jacoupy M. Nicolas A. (2016). Loss of VPS13C function in autosomal-recessive parkinsonism causes mitochondrial dysfunction and increases PINK1/Parkin-dependent mitophagy. *Am. J. Hum. Genet.* 98 500–513. 10.1016/j.ajhg.2016.01.014 26942284 PMC4800038

[B57] Lewis S. C. Uchiyama L. F. Nunnari J. (2016). ER-mitochondria contacts couple mtDNA synthesis with mitochondrial division in human cells. *Science* 353:aaf5549. 10.1126/science.aaf5549 27418514 PMC5554545

[B58] Lim Y. Cho I. T. Schoel L. J. Cho G. Golden J. A. (2015). Hereditary spastic paraplegia-linked REEP1 modulates endoplasmic reticulum/mitochondria contacts. *Annal. Neurol.* 78 679–696. 10.1002/ana.24488 26201691 PMC4681538

[B59] Lynes E. M. Bui M. Yap M. C. Benson M. D. Schneider B. Ellgaard L. (2012). Palmitoylated TMX and calnexin target to the mitochondria-associated membrane. *EMBO J.* 31 457–470. 10.1038/emboj.2011.384 22045338 PMC3261551

[B60] Manor U. Bartholomew S. Golani G. Christenson E. Kozlov M. Higgs H. (2015). A mitochondria-anchored isoform of the actin-nucleating spire protein regulates mitochondrial division. *ELife* 4:e08828. 10.7554/eLife.08828 26305500 PMC4574297

[B61] Meisinger C. Wiedemann N. Rissler M. Strub A. Milenkovic D. Schönfisch B. (2006). Mitochondrial protein sorting: differentiation of β-barrel assembly by tom7-mediated segregation of Mdm10. *J. Biol. Chem.* 281 22819–22826. 10.1074/jbc.M602679200 16760475

[B62] Modi S. López-Doménech G. Halff E. F. Covill-Cooke C. Ivankovic D. Melandri D. (2019). Miro clusters regulate ER-mitochondria contact sites and link cristae organization to the mitochondrial transport machinery. *Nat. Commun.* 10:4399. 10.1038/s41467-019-12382-4 31562315 PMC6764964

[B63] Moser von Filseck J. Čopič A. Delfosse V. Vanni S. Jackson C. L. Bourguet W. (2015). Phosphatidylserine transport by ORP/Osh proteins is driven by phosphatidylinositol 4-phosphate. *Science* 349 432–436. 10.1126/science.aab1346 26206936

[B64] Muñoz-Braceras S. Calvo R. Escalante R. (2015). TipC and the chorea-acanthocytosis protein VPS13A regulate autophagy in *Dictyostelium* and human HeLa cells. *Autophagy* 11 918–927. 10.1080/15548627.2015.1034413 25996471 PMC4507429

[B65] Muñoz-Braceras S. Tornero-Écija A. R. Vincent O. Escalante R. (2019). VPS13A is closely associated with mitochondria and is required for efficient lysosomal degradation. *Dis. Model. Mech.* 12:dmm036681. 10.1242/DMM.036681 30709847 PMC6398486

[B66] Murley A. Lackner L. L. Osman C. West M. Voeltz G. K. Walter P. (2013). ER-associated mitochondrial division links the distribution of mitochondria and mitochondrial DNA in yeast. *ELife* 2:e00422. 10.7554/eLife.00422 23682313 PMC3654481

[B67] Nagashima S. Takeda K. Ohno N. Ishido S. Aoki M. Saitoh Y. (2019). MITOL deletion in the brain impairs mitochondrial structure and ER tethering leading to oxidative stress. *Life Sci. Alliance* 2:e201900308. 10.26508/lsa.201900308 31416892 PMC6696985

[B68] Naon D. Zaninello M. Giacomello M. Varanita T. Grespi F. Lakshminaranayan S. (2016). Critical reappraisal confirms that Mitofusin 2 is an endoplasmic reticulum-mitochondria tether. *Proc. Natl. Acad. Sci. U S A.* 113 11249–11254. 10.1073/pnas.1606786113 27647893 PMC5056088

[B69] Nguyen T. T. Lewandowska A. Choi J. Y. Markgraf D. F. Junker M. Bilgin M. (2012). Gem1 and ERMES do not directly affect phosphatidylserine transport from ER to mitochondria or mitochondrial inheritance. *Traffic* 13 880–890. 10.1111/j.1600-0854.2012.01352.x 22409400 PMC3648210

[B70] Patron M. Checchetto V. Raffaello A. Teardo E. VecellioReane D. Mantoan M. (2014). MICU1 and MICU2 finely tune the mitochondrial Ca^2+^ uniporter by exerting opposite effects on MCU activity. *Mol. Cell.* 53 726–737. 10.1016/j.molcel.2014.01.013 24560927 PMC3988891

[B71] Petrungaro C. Kornmann B. (2019). Lipid exchange at ER-mitochondria contact sites: a puzzle falling into place with quite a few pieces missing. *Curr. Opin. Cell. Biol.* 57 71–76. 10.1016/j.ceb.2018.11.005 30554079

[B72] Petrungaro C. Zimmermann K. M. Küttner V. Fischer M. Dengjel J. Bogeski I. (2015). The Ca^2+^-dependent release of the Mia40-induced MICU1-MICU2 dimer from MCU regulates mitochondrial Ca^2+^ uptake. *Cell Metabol.* 22 721–733. 10.1016/j.cmet.2015.08.019 26387864

[B73] Prudent J. Zunino R. Sugiura A. Mattie S. Shore G. C. McBride H. M. (2015). MAPL SUMOylation of Drp1 stabilizes an ER/mitochondrial platform required for cell death. *Mol. Cell.* 59 941–955. 10.1016/j.molcel.2015.08.001 26384664

[B74] Puri R. Cheng X. T. Lin M. Y. Huang N. Sheng Z. H. (2019). Mul1 restrains Parkin-mediated mitophagy in mature neurons by maintaining ER-mitochondrial contacts. *Nat. Comm.* 10. 10.1038/s41467-019-11636-5 31409786 PMC6692330

[B75] Rapizzi E. Pinton P. Szabadkai G. Wieckowski M. R. Vandecasteele G. Baird G. (2002). Recombinant expression of the voltage-dependent anion channel enhances the transfer of Ca^2+^ microdomains to mitochondria. *J. Cell Biol.* 159 613–624. 10.1083/jcb.200205091 12438411 PMC2173108

[B76] Raturi A. Gutiérrez T. Ortiz-Sandoval C. Ruangkittisakul A. Herrera-Cruz M. S. Rockley J. P. (2016). TMX1 determines cancer cell metabolism as a thiolbased modulator of ER-mitochondria Ca^2+^ flux. *J. Cell Biol.* 214 433–444. 10.1083/jcb.201512077 27502484 PMC4987292

[B77] Rizzuto R. Brini M. Murgia M. Pozzan T. (1993). Microdomains with high Ca^2+^ close to IP3-sensitive channels that are sensed by neighboring mitochondria. *Science* 262 744–747. 10.1126/science.8235595 8235595

[B78] Rochin L. Sauvanet C. Jääskeläinen E. Houcine A. Kivelä A. Xingjie M. A. (2019). Orp5 transfers phosphatidylserine to mitochondria and regulates mitochondrial calcium uptake at endoplasmic reticulum - mitochondria contact sites. *bioRxiv* 2019:695577. 10.1101/695577

[B79] Roth D. Lynes E. Riemer J. Hansen H. G. Althaus N. Simmen T. (2009). A di-arginine motif contributes to the ER localization of the type I transmembrane ER oxidoreductase TMX4. *Biochem. J.* 425 195–208. 10.1042/BJ20091064 19811453

[B80] Rouzier C. Moore D. Delorme C. Lacas-Gervais S. Ait-El-Mkadem S. Fragaki K. (2017). A novel CISD2 mutation associated with a classical wolfram syndrome phenotype alters Ca^2+^ homeostasis and ER-mitochondria interactions. *Hum. Mol. Genet.* 26 1599–1611. 10.1093/hmg/ddx060 28335035 PMC5411739

[B81] Schauder C. M. Wu X. Saheki Y. Narayanaswamy P. Torta F. Wenk M. R. (2014). Structure of a lipid-bound extended synaptotagmin indicates a role in lipid transfer. *Nature* 510 552–555. 10.1038/nature13269 24847877 PMC4135724

[B82] Scorrano L. De Matteis M. A. Emr S. Giordano F. Hajnóczky G. Kornmann B. (2019). Coming together to define membrane contact sites. *Nat. Commun.* 10:1287. 10.1038/s41467-019-09253-3 30894536 PMC6427007

[B83] Shai N. Yifrach E. Van Roermund C. W. T. Cohen N. Bibi C. Ijlst L. (2018). Systematic mapping of contact sites reveals tethers and a function for the peroxisome-mitochondria contact. *Nat. Commun.* 9:1761. 10.1038/s41467-018-03957-8 29720625 PMC5932058

[B84] Shirane M. Wada M. Morita K. Hayashi N. Kunimatsu R. Matsumoto Y. (2020). Protrudin and PDZD8 contribute to neuronal integrity by promoting lipid extraction required for endosome maturation. *Nat. Commun.* 11:4576. 10.1038/s41467-020-18413-9 32917905 PMC7486383

[B85] Stoica R. De Vos K. J. Paillusson S. Mueller S. Sancho R. M. Lau K. F. (2014). ER-mitochondria associations are regulated by the VAPB-PTPIP51 interaction and are disrupted by ALS/FTD-associated TDP-43. *Nat. Commun.* 5:3996. 10.1038/ncomms4996 24893131 PMC4046113

[B86] Stroud D. A. Oeljeklaus S. Wiese S. Bohnert M. Lewandrowski U. Sickmann A. (2011). Composition and topology of the endoplasmic reticulum-mitochondria encounter structure. *J. Mol. Biol.* 413 743–750. 10.1016/j.jmb.2011.09.012 21945531

[B87] Sugiura A. Nagashima S. Tokuyama T. Amo T. Matsuki Y. Ishido S. (2013). MITOL regulates endoplasmic reticulum-mitochondria contacts via Mitofusin2. *Mol. Cell* 51 20–34. 10.1016/j.molcel.2013.04.023 23727017

[B88] Szabadkai G. Bianchi K. Várnai P. De Stefani D. Wieckowski M. R. Cavagna D. (2006). Chaperone-mediated coupling of endoplasmic reticulum and mitochondrial Ca^2+^ channels. *J. Cell Biol.* 175 901–911. 10.1083/jcb.200608073 17178908 PMC2064700

[B89] Valm A. M. Cohen S. Legant W. R. Melunis J. Hershberg U. Wait E. (2017). Applying systems-level spectral imaging and analysis to reveal the organelle interactome. *Nature* 546 162–167. 10.1038/nature22369 28538724 PMC5536967

[B90] Vance J. E. (1990). Phospholipid synthesis in a membrane fraction associated with mitochondria. *J. Biol. Chem.* 265 7248–7256. 10.1016/S0021-9258(19)39106-9 2332429

[B91] Wang C. H. Chen Y. F. Wu C. Y. Wu P. C. Huang Y. L. Kao C. H. (2014). Cisd2 modulates the differentiation and functioning of adipocytes by regulating intracellular Ca^2+^ homeostasis. *Hum. Mol. Genet.* 23 4770–4785. 10.1093/hmg/ddu193 24833725

[B92] Wang P. T. C. Garcin P. O. Fu M. Masoudi M. St-Pierre P. Panté N. (2015). Distinct mechanisms controlling rough and smooth endoplasmic reticulum contacts with mitochondria. *J. Cell Sci.* 128 2759–2765. 10.1242/jcs.171132 26065430

[B93] Wong Y. C. Ysselstein D. Krainc D. (2018). Mitochondria-lysosome contacts regulate mitochondrial fission via RAB7 GTP hydrolysis. *Nature* 554 382–386. 10.1038/nature25486 29364868 PMC6209448

[B94] Wu H. Carvalho P. Voeltz G. K. (2018). Here, there, and everywhere: the importance of ER membrane contact sites. *Science* 361 eaan5835. 10.1126/science.aan5835 30072511 PMC6568312

[B95] Wu S. Lu Q. Wang Q. Ding Y. Ma Z. Mao X. (2017). Binding of FUNDC1 with inositol 1,4,5-trisphosphate receptor in mitochondria-associated endoplasmic reticulum (ER) membranes maintains mitochondrial dynamics and function in hearts in vivo. *Circulation* 136 2248–2266. 10.1161/CIRCULATIONAHA.117.030235 28942427 PMC5716911

[B96] Wu W. Lin C. Wu K. Jiang L. Wang X. Li W. (2016). FUNDC 1 regulates mitochondrial dynamics at the ER –mitochondrial contact site under hypoxic conditions. *EMBO J.* 35 1368–1384. 10.15252/embj.201593102 27145933 PMC4864280

[B97] Xu L. Wang X. Zhou J. Qiu Y. Shang W. Liu J. P. (2020). Miga-mediated endoplasmic reticulum– mitochondria contact sites regulate neuronal homeostasis. *ELife* 9 1–26. 10.7554/eLife.56584 32648543 PMC7556861

[B98] Yamano K. Tanaka-Yamano S. Endo T. (2010). Tom7 regulates Mdm10-mediated assembly of the mitochondrial import channel protein TOM40. *J. Biol. Chem.* 285 41222–41231. 10.1074/jbc.M110.163238 21036907 PMC3009848

[B99] Yeshaw W. M. van der Zwaag M. Pinto F. Lahaye L. L. Faber A. I. E. Gómez-Sánchez R. (2019). Human VPS13A is associated with multiple organelles and influences mitochondrial morphology and lipid droplet motility. *ELife* 8 1–37. 10.7554/eLife.43561 30741634 PMC6389287

